# Intersectoral collaboration in the management of non-communicable disease’s risk factors in Iran: stakeholders and social network analysis

**DOI:** 10.1186/s12889-022-14041-8

**Published:** 2022-09-02

**Authors:** Ahad Bakhtiari, Amirhossein Takian, Reza Majdzadeh, Afshin Ostovar, Mehdi Afkar, Narges Rostamigooran

**Affiliations:** 1 Health Equity Research Centre (HERC), Tehran University of Medical Sciences, Tehran, Iran; 2 Department of Global Health and Public Policy, School of Public Health, Tehran University of Medical Sciences, Tehran, Iran; 3 Department of Health Management, Policy and Economics, School of Public Health, Tehran University of Medical Sciences, Tehran, Iran; 4 Department of Epidemiology and Biostatistics, School of Public Health, Tehran University of Medical Sciences, Tehran, Iran; 5 Osteoporosis Research Center, Endocrinology and Metabolism Clinical Sciences Institute, Tehran University of Medical Sciences, Tehran, Iran; 6 Center for Non-Communicable Disease Control, Ministry of Health and Medical Education, Tehran, Iran; 7 Secretariat of Supreme Council of Health and Food Security, Ministry of Health and Medical Education, Tehran, Iran

**Keywords:** Noncommunicable diseases (NCDs), Risk factors, Supreme councils, Social network analysis (SNA), Intersectoral collaboration (ISC)

## Abstract

**Introduction:**

As the major cause of premature death worldwide, noncommunicable diseases (NCDs) are complex and multidimensional, prevention and control of which need global, national, local, and multisectoral collaboration. Governmental stakeholder analysis and social network analysis (SNA) are among the recognized techniques to understand and improve collaboration. Through stakeholder analysis, social network analysis, and identifying the leverage points, we investigated the intersectoral collaboration (ISC) in preventing and controlling NCDs-related risk factors in Iran.

**Methods:**

This is a mixed-methods study based on semi-structured interviews and reviewing of the legal documents and acts to identify and assess the interest, position, and power of collective decision-making centers on NCDs, followed by the social network analysis of related councils and the risk factors of NCDs. We used Gephi software version 0.9.2 to facilitate SNA. We determined the supreme councils' interest, position, power, and influence on NCDs and related risk factors. The Intervention Level Framework (ILF) and expert opinion were utilized to identify interventions to enhance inter-sectoral collaboration.

**Results:**

We identified 113 national collective decision-making centers. Five councils had the highest evaluation score for the four criteria (Interest, Position, Power, and Influence), including the Supreme Council for Health and Food Security (SCHFS), Supreme Council for Standards (SCS), Supreme Council for Environmental Protection (SCIP), Supreme Council for Health Insurance (SCHI) and Supreme Council of the Centers of Excellence for Medical Sciences. We calculated degree, in degree, out-degree, weighted out-degree, closeness centrality, betweenness centrality, and Eigenvector centrality for all councils. Supreme Council for Standards and SCHFS have the highest betweenness centrality, showing Node's higher importance in information flow. Interventions to facilitate inter-sectoral collaboration were identified and reported based on Intervention Level Framework's five levels (ILF).

**Conclusion:**

A variety of stakeholders influences the risk factors of non-communicable diseases. Through an investigation of stakeholders and their social networks, we determined the primary actors for each risk factor. Through the different (levels and types) of interventions identified in this study, the MoHME can leverage the ability of identified stakeholders to improve risk factors management. The proposed interventions for identified stakeholders could facilitate intersectoral collaboration, which is critical for more effective prevention and control of modifiable risk factors for NCDs in Iran. Supreme councils and their members could serve as key hubs for implementing targeted inter-sectoral approaches to address NCDs' risk factors.

**Supplementary Information:**

The online version contains supplementary material available at 10.1186/s12889-022-14041-8.

## Background

NCDs have been identified as the leading cause of Years of Life Lost (YLL), Disability-Adjusted Life Years (DALYs), and mortality in countries by controlling communicable diseases, changing lifestyles, and growing risk factors for NCDs such as air pollution, low physical activity, and unhealthy nutrition. The share of NCDs from total deaths, both sexes, all ages increased between 1990 and 2019 in Iran (50.11% to 83.48%), the world (57.72% to 74.37%); a similar trend has occurred in the share of NCDs from total DALYs, Iran (45.33% to 78.09%), the world (43.19% to 63.82%); the global economic burden of NCDs is predicted to be USD47 trillion between 2010 and 2030 [1–4]. According to the Global Burden of Disease (GBD) 2019, about 16.5%, 4.5%, 18.8%, 30.6%, and 14.1% of all deaths in Iran in 2019 were attributable to dietary risk factors, insufficient physical activity (IPA), high BMI, high systolic blood pressure, and tobacco use respectively. The Iran STEPs 2016 survey revealed a high prevalence of IPA, approximately 54.7% of the total population, as defined by WHO recommendation, which is less than 600 Metabolic Equivalent of Task (MET) per week, and 9.6% of participants were current cigarette smokers, the majority of whom were aged 45–70 [5, 6]. Between 2011 and 2025, monetary losses to the low and middle-income countries (LMICs) that emerged from four main NCDs are predicted to exceed US$ 7 trillion, 70% of which will occur in the upper-middle-income countries (UMICs) [7]. It has been determined that 51% of this yearly loss will be linked to cardiovascular diseases, equal to around 4% of these countries’ current annual output [7]. Let alone that the COVID-19 pandemic might exacerbate NCDs' burden due to possible ignorance of NCDs’ priority [8].

NCDs’ prevention and control need global, national, multisectoral, and multistakeholder engagement. As a key strategy to achieve health systems’ goals and reduce health inequities, inter-sectoral collaboration is recommended by the “Global Action Plan” of the World Health Organization (WHO) for the prevention and control of NCDs, as well as by the “National Plan for Prevention and Control of NCDs” in Iran [3, 9, 10]. Nevertheless, the status of current inter-sectoral collaboration initiatives in Iran does not appear as meaningful as they are supposed to be for effective management of NCDs [11], e.g., in the field of urban and transport planning [12], food industry [13].

Meaningful prevention and control of NCDs need a comprehensive approach, which brings all sectors on board to work unitedly to reduce the risks associated with NCDs and to prevent and control NCDs' burden on communities. These include, but are not limited to, health, economics, diplomacy, foreign health policy, education system, agriculture, insurance organizations, markets, tax system, food industry, legislative system, etc. [14–17]. Intersectoral collaboration (ISC) is mentioned in Articles VII and VIII of the Alma Ata Declaration of 1978: *“All governments should formulate national policies, strategies, and plans of action to launch, and sustain primary health care as part of a comprehensive national health system and in coordination with other sectors. To this end, it will be necessary to exercise political will, to mobilize the country's resources, and to use available external resources rationally”* [18].

WHO also issued another statement, so-called Intersectoral Action for Health (IAH), which means *“a recognized relationship between part or parts of the health sector with parts of another sector which has been formed to take action on an issue to achieve health outcomes (or intermediate health outcomes) in a way that is more effective, efficient or sustainable than could be achieved by the health sector acting alone”* [19]. Other global statements, including Primary Health Care (PHC) [20], Millennium Development Goals (MDG) [21], and Sustainable Development Goals (SDGs) also consider ISC as one of the most necessary principles for the health system [22–26].

Stakeholder analysis facilitates the classification of stakeholders, valuing and comparing their particular sets of interests and powers, and examining and reviewing their relationships, including alliances, collaborations, and inherent conflicts [27]. It investigates “who these interested parties are, who has the power to influence what happens, how these parties interact and, based on this information, how they might be able to work more effectively together” [28]. Social network analysis (SNA) is a powerful technique for analyzing the relationship among different actors/stakeholders/parts of government and understanding possible ways to improve collaboration towards better outcomes. The insights gained from SNA can help coordinate ISC global and national efforts and interventions for more efficient prevention and control of NCDs [29], i.e., those recommended by the WHO’s PEN (Package of essential NCDs interventions) [9]. Social network theory and SNA help researchers explain the relationship between people, organizations, or even nations, which might enable them to explore the existing connections and draw a more realistic picture of underlying relationships among them [30]. Visualizing ISC as networks can also facilitate stakeholder network analysis techniques and network theories to discover how they can be more effective in tackling NCDs [29, 31].

Embedding collective decision-making mechanisms into the national administrative system is the key to successfully implementing policies in all settings [32]. Iran enjoys several collective decision-making bodies. So-called the “Councils” and the “Supreme Councils” support coordination, policymaking, and planning of joint decisions, taking which requires ISC among various entities, i.e., different ministries in different areas such as health, economy, welfare, the judicial system, and so on [33]. So-called the “cabinet committee”, such a mechanism exists in other countries; a group made up of cabinet ministers, which is formed to enable meaningful actions on a particular issue or general area of importance for the government [34]. Opportunities for ISC between the expert levels are also available in these councils.

The study’s objectives and questions are: What collective decision-making centers exist at the national level to progress IAH and ISC?; Which policies of identified stakeholders influence risk factors?; What interventions (at different levels) can be put on the agenda to control better the policies that have been approved?. Our findings will support, we envisage, national policymaking on NCDs and related risk factors for better knowledge translation and implementing laws and standards in Iran and perhaps similar settings.

## Methods

This is a mixed-methods study based on reviewing legal documents and acts and conducting semi-structured interviews to identify and evaluate the interest, position, and power of collective decision-making centers (councils) and their members on NCDs, followed by the Social Network Analysis (SNA). We used Gephi software [35] version 0.9.2 to facilitate SNA. In the final step, based on the Intervention Level Framework (ILF), we identified key leverage points for maximizing the capacity of the identified stakeholders for ISC.

### Understanding and identification of NCDs, risk factors of NCDs, and their relationship

To identify major risk factors of NCDs, we used WHOs' 5 × 5 matrix, i.e., five NCDs and five modifiable shared risk factors [5], 'best buys' and other recommended interventions for the prevention and control of NCDs [36, 37], the global action plan for the prevention and control of NCDs 2013–2020 [38] plus the GBD tools developed by the “Institute for Health Metrics and Evaluation” (IHME) [39], which includes 359 diseases and injuries and 84 risk factors. We mapped the network among the risk factors and diseases based on the burden of NCDs and related risk factors. Each risk factor's contribution to shaping the burden (DALY) of NCDs in Iran was obtained from the GBD study of 2017, whose weight and diameter were reflected in the nodes and edges.

### Identification of councils and their details

#### Reviewing of documents

National councils are the place for collective decision-making among relevant ministries and other institutions. We defined the search query to identify the councils. Keywords ( Supreme Council or Supreme Councils or Board of Examiner or Crossbreed Councils or Administrative Council or Administrative Councils or Strategic Council or Strategic Councils or Board of Directors or High Representative or Strategic Council or Strategic Councils or Commission or Committee or Headquarters or Workgroup or Board or Council) were searched in the country's comprehensive law databases (National System of Laws (https://qavanin.ir/Law), parliament system of laws (https://rc.majlis.ir/en); Ministry of Health system of laws (https://healthcode.behdasht.gov.ir/approvals/)), from 1941 until 2020.

#### Interviews

Semi-structured face-to-face interviews with purposefully selected experts (Appendix B.2) were conducted to properly identify the stakeholders and understand their roles. The interviews, which lasted between 20 and 70 min each, were conducted between August 2020 and December 2020, to ensure data saturation. All interviews occurred at the interviewees' workplaces. The researchers conducted the interviews using a literature-based and designed interview guide. The following questions were explored during the interviews: Which actors and decision-making institutions are involved in ISC for NCDs' risk factors? Which dimensions and risk factors are affected by their policies? How do you perceive their responsibilities, abilities, motivations, effects, and interactions? What are your recommendations or interventions for enhancing ISC? The interviews were transcribed verbatim, and the data were analyzed using a thematic content analysis.

The councils' statutes, mission, objectives, members, and organization were extracted following their identification. We created a list of each council's resolutions since its establishment using the databases mentioned and their official website (Table 1).

**Table 1:** Data collection structure for each council

Councils	Mission and objectives	Members	Structure	Approved resolutions
1. X	- Health-oriented objectives - Objectives with an indirect relationship with health - Unrelated health objectives	- The president - Ministries - Organizations - MoHME	- Position in the structure of authority - Chairman of the council - Secretariat - Committees	- Health-oriented resolutions - Resolutions with an indirect relationship with health -Unrelated health resolutions

Each council's interest, position, power, and influence were valued by reviewing its mission and objectives, members, structure, and resolutions (Table 2).

**Table 2:** Definitions and scoring of the criteria of each council

Council	Definition	Scoring
Interest	As the number of approved resolutions related to NCDs^a^	Low, low-medium, medium, medium–high, and high, 0–5 / 6–8 / 9–12/ 13–15 and more than 15, respectively
Position	As a council that outlined a health-based mission and objectives in its statute, the MoHME had a significant role in guiding it	Low, low-medium, medium, medium–high, and high
Power	As a statutory authority given to the council in the statute to deal with NCDs and related risk factors	Low, low-medium, medium, medium–high, and high
Influence	The number of risk factors that can be influenced by the council	The number of risk factors that the council has the potential to influence

^a^ *NCDs* Non-Communicable Diseases

We used qualitative textual evidence to identify the councils with potential impact on NCDs’ risk factors. These were official public documents [40], to which we had direct or indirect access, and were therefore credible [41]. We considered NCDs and related risk factors as the main framework for searching for relevant councils to sketch a network of NCDs, risk factors, and councils. Based on each council's documents' analysis, the council's potential connection with each risk factor was defined.

#### Generating the network and statistical analysis

Following the identification of stakeholders (Supreme Councils and their members), the network's elements were defined (Table 3) [9, 10, 42–46]. Members of councils, councils, risk factors, NCDs and related SDGs were defined as nodes and connections among them were considered as edges (weighted and directed).

**Table 3:** Network elements

Network elements as nodes	Nodes based on	Edges based on	Target
Members	- Ministry: strong -Government organization: medium -Private Sector and NGOs^a^: poor	-Has the right to vote in the Supreme Council: Strong -No voting rights: Poor	Councils
Councils	Based on Table 1	Based on Table 1	Risk factors
SDGs^b^	Based on seven selected studies and reports [39–45]	Based on seven studies and reports	Risk factors and NCDs
Risk factors	Attribute DALY in 2017	Attribute DALY in 2017	NCDs

^a^ *NGOs* Non-Governmental Organization, ^b^ *SDGs* Sustainable Development Goals, *NCDs* Non-Communicable Diseases

Networks present a natural approach to showing social [47] or information systems [26]. We used Gephi software [35], version 0.9.2, for analyzing and displaying networks graphically. The NCDs stakeholder network was statistically examined concerning associative depth and associative clustering with Gephi software. These statistical characteristics were determined by applying different algorithms with Gephi. We selected the “Mike Bostock circle packing algorithm” [26]. We carried out analyses including degree, in degree, out-degree, weighted out-degree, closeness centrality, betweenness centrality, hub, authority and eigenvector centrality. (Please see Appendix A for more information on the terms). Finally, we asked a few key experts to approve our findings.

#### Recognizing the leverage points to make more use of the power of the stakeholders

**Fig. 1:**
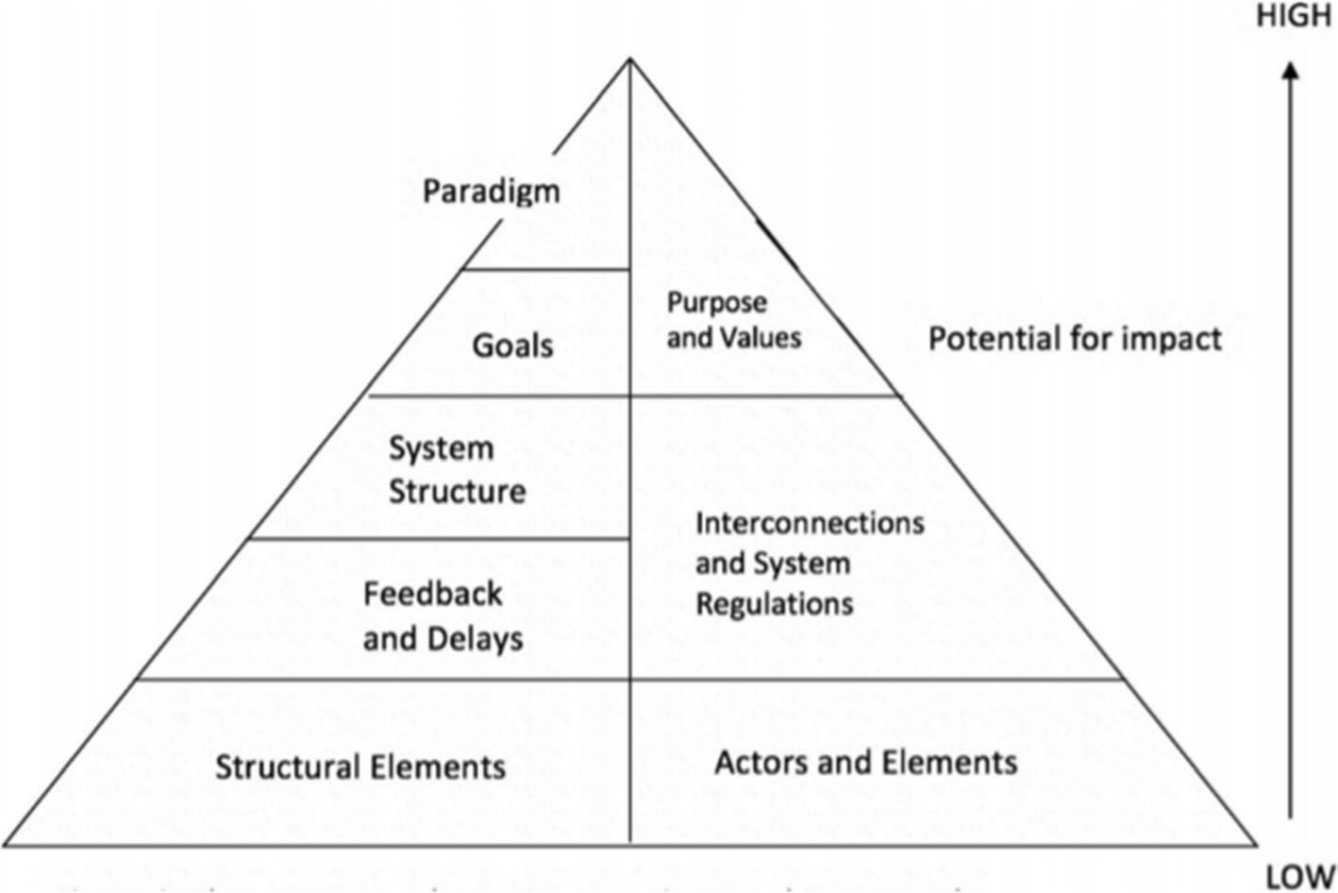
The Intervention Level Framework (ILF)

“Leverage points”, defined as places within a complex system (a large company, economy, living body, city, ecosystem), where a small change in one point can make a big difference in the whole system, are important in system analysis. Identifying where leverage points are and how they can be reached is crucial to determining the points of power, as introduced in the Intervention Level Framework (ILF) (Fig. 1), as well as other complementary models of leverage points [48]. This model classifies the dimensions of the intervention level framework that can be used for reforms, whose aim is to increase the role of the Ministry of Health in the councils with an impact on population health.

#### Initial identification of interventions

On the basis of the interview results, initial interventions were determined. Document review was used to complete the initial list of interventions; all council documents (statutes, objectives, laws, structure, members, committees, approvals, processes, and reports, i.e. both official and grey literature) were collected and reviewed. The study team listed the initial interventions at different ILF levels and held several sessions to review and summarize the previous steps' results.

#### Identification of final interventions

We obtained the viewpoints of 33 experts (Appendix B) using an online questionnaire containing the initial list of interventions. Moreover, we sought the experts’ views on the appropriateness and feasibility of the proposed interventions (Appendix B). All study steps were performed in accordance with the relevant ethical and research guidelines and regulations; and supervised by the ethics committee of Tehran University of Medical Sciences.

## Results

We identified 113 national collective decision-making centers in the Islamic Republic of Iran's administrative and legal system. Following the initial screening, 21 cases went into the next steps to identify each council's effect on NCDs’ risk factors. These were the collective decision-making actors that were able to influence NCDs. All 21 councils were initiated within the government and had governmental and non-governmental members. Nevertheless, all members and non-members of the councils are obliged to observe these councils' acts. The majority of the 50 members of these councils are from the ministries, while only seven members (14%) are non-governmental. We did not map out individual stakeholders, for example, honorary membership without the right to vote.

**Fig. 2:**
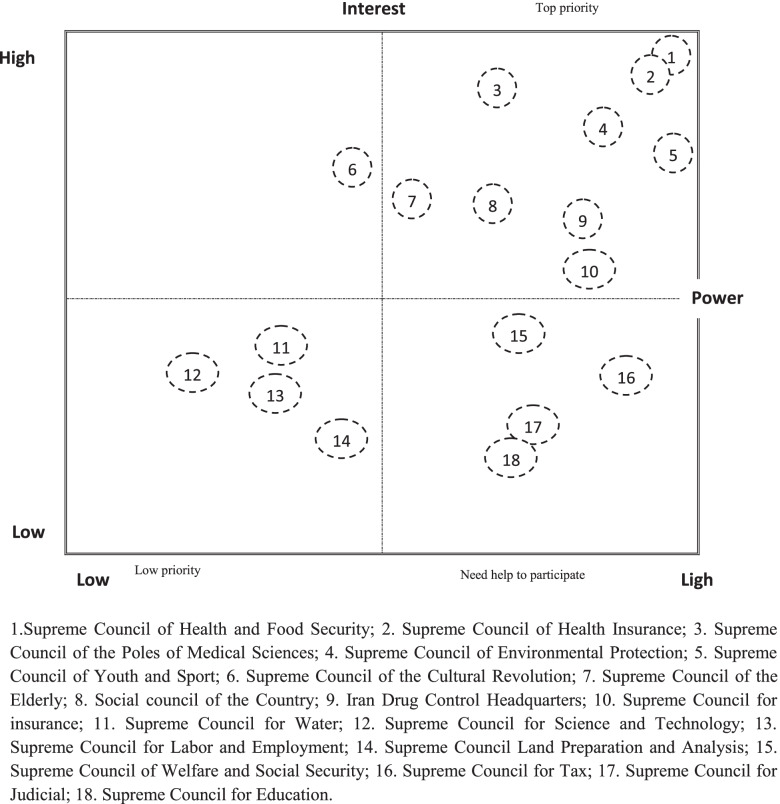
Matrix of the councils based on their interest and power

Estimated criteria, interest, position, power, and influence of key councils are shown in Table 4. The five councils had the highest evaluation score in the four criteria, including the Supreme Council of Health and Food Security, Supreme Council for Standards, Supreme council for Environmental Protection, Supreme Council of Health Insurance, and Supreme Council for Centers of Excellence in Medical Sciences of Medical Sciences. The matrix of councils based on their interest and power has also been analyzed and mapped (Fig. 2).

**Table 4:** Councils and Stakeholders involved with the process of NCDs-related policymaking in Iran rated according to four items (interest, position, power, and influence)

	**Number of Stakeholders (Members)**	**MoHME** **(Has a leadership role in the council: a green label)**	**Interest**	**Position**	**Power**	**Influence (on)**	**Type of influence**
1. Supreme Council of Health and Food Security	16^a^	Yes	High	High	High	**High,** Dietary risk, Second-hand smoke, Drug use, Smoking, Chewing tobacco, Alcohol use, High fasting plasma glucose, High body mass index, High systolic blood pressure, High LDL cholesterol, Ambient ozone pollution, Occupational risks, Lead exposure, Ambient particulate matter air pollution, Impaired kidney function	Legislation, research, inter-sectoral cooperation, awareness
2. Supreme Council for Justice	10	No	Low	Low	Medium	**Medium–high,** Dietary risk, Second-hand smoke, Drug use, Smoking, Chewing tobacco, Alcohol use, Ambient ozone pollution, Occupational risks, Lead exposure, Ambient particulate matter air pollution, Occupational risks	Creating or amending judicial laws
3. Supreme Council for Centers of Excellence in Medical Sciences	15	Yes	High	High	Medium	**High,** Dietary risk, Second-hand smoke, Drug use, Smoking, Chewing tobacco, Alcohol use, High fasting plasma glucose, High body mass index, High systolic blood pressure, High LDL cholesterol, Ambient ozone pollution, Occupational risks, Ambient particulate matter air pollution, Impaired kidney function	Specialized research in the field of each risk factor
4. Supreme Council for Youth & Sports	18	Yes	Medium–high	Medium–high	High	**Medium,** Dietary risk, Second-hand smoke, Drug use, Smoking, Chewing tobacco, Alcohol use, High body mass index High, systolic blood pressure	Expansion of public sports facilities, awareness among youth about risk factors
5. Supreme Council for Standards	29^b^	Yes	High	High	High	**High,** Dietary risk, Second-hand smoke, Drug use, Smoking, Chewing tobacco, Alcohol use, High fasting plasma glucose, High body mass index, High systolic blood pressure, High LDL cholesterol, Ambient ozone pollution, Occupational risks, Lead exposure, Ambient particulate matter air pollution	Create or modify standards for sources of risk factors
6. Supreme Council for Water	10	No	Medium	Low-medium	Medium	**Low,** Dietary risk	Water policy for food production
7. Supreme Council for Education	15	No	Low	Low	Medium	**Medium,** Dietary risk, Second-hand smoke, Drug use, Smoking, Chewing tobacco, Alcohol use, High body mass index, High systolic blood pressure,	Educate students and incorporate health-oriented lessons into their courses
8. Supreme Council for Insurance	11	No	Medium–high	Low-medium	Medium–high	**Low,** Dietary risk	Provision of minimum salaries for retirement and sickness
9. Supreme Council of Health Insurance	14	Yes	High	High	High	**Medium–high,** Drug use, Smoking, Alcohol use, High fasting plasma glucose, High body mass index, High systolic blood pressure, High LDL cholesterol, Occupational risks, Lead exposure, and Impaired kidney function	Preventive services coverage Set franchise Covering the uninsured and the poor
10. Supreme Council of the Cultural Revolution	43	Yes	Medium–high	Medium	Medium	**Medium,** Dietary risk, Second-hand smoke, Drug use, Smoking, Chewing tobacco, Alcohol use, and High body mass index	The main cultural policymaker in Iran
11. Supreme Council for Science, Research & Technology	24	Yes	Low-medium	Medium	Low-medium	**Low-medium**, Ambient ozone pollution, Occupational risks, Lead exposure	Research, especially in the field of industry
12. Supreme Council for Environmental Protection	13	Yes	High	High	High	**Medium–high,** Dietary risk, Ambient ozone pollution, Occupational risks, Lead exposure, Ambient particulate matter air pollution	The main policymaker in the field of soil, air, and water pollutants
13. Supreme Council of Welfare and Social Security	13	Yes	Medium–high	Medium–high	Medium–high	**Low,** Dietary risk	Welfare Policy Making
14. Supreme Council for Labor and Employment	22	Yes	Low-medium	Medium	Medium	**Low-medium,** Dietary risk, Occupational risks	Employment promotion and determination of workers' rights and working conditions
15. Supreme Council for Tax	25	No	Low-medium	Low-medium	Medium–high	**Medium–high,** Dietary risk, Second-hand smoke, Drug use, Smoking, Chewing tobacco, Alcohol use, Ambient ozone pollution, Occupational risks, Lead exposure, Ambient particulate matter air pollution	Determining tax policies in the area of risk factors resources
16. Supreme Council for Urbanization	14	No	Low-medium	Medium	Medium–high	**Medium,** High body mass index, Ambient particulate matter, air pollution, Lead exposure	Policymakers to build healthy cities in terms of physical activity, pollution, etc
17. High Council for Land Preparation and Analysis	20	No	Low	Medium	Medium	**Low,** Dietary, Ambient particulate matter air pollution	The exponential location of the creation of cities, agricultural areas, Estimating the reception capacity of the population of the regions
18. National Council for the Elderly	18	Yes	Medium–high	High	Medium	**High,** Dietary risk, Second-hand smoke, Drug use, Smoking, Chewing tobacco, Alcohol use, High fasting plasma glucose, High body mass index, High systolic blood pressure, High LDL cholesterol	Determining welfare and health policies for the elderly
19. Social Council of the country	26	Yes	Medium–high	Medium–high	Medium–high	**Medium,** Dietary risk, Second-hand smoke, Drug use, Smoking, Chewing tobacco, Alcohol use	Fighting social harm and protecting people exposed to social harm
20. Iran Drug Control Headquarters	12	Yes	Medium–high	Medium–high	High	**Medium**, Second-hand smoke, Drug use, Smoking, Chewing tobacco, Alcohol use	Policy on tobacco, alcohol, and drugs
21. Hygienic monitoring and control committee on toxins and chemicals	6	Yes	Medium–high	High	High	**Medium,** Dietary risk, Occupational risks, Lead exposure	Main policymaking in the field of agricultural pesticides and hazardous chemicals

^a^ 1. President (Chairman); 2- Minister of Health and Medical Education (Secretary); 3. Head of the Country Management and Planning Organization; 4. Minister of Education; 5. Minister of Agriculture Jihad; 6. The Minister of Commerce; 7. Minister of Industry, Mine, and Trade; 8. Minister of Cooperatives, Labour, and Social Welfare; 9. Minister of the Interior; 10. Minister of Justice; 11. Minister of Energy; 12. Minister of the case under review; 13- Head of IRIB; 14. Head of Department of Environment; 15. Head of Physical Education Organization; 16- Head of the Medical Council of Iran

^b^ 1. President as Chairman; 2. Head of National Standard Organization as Secretary; 3. Head of Management and Planning Organization of Iran; 4. Head of Iranian Department of Environment; 5. Ministers of Economic Affairs and Finance; 6.Ministry of Science, Research and Technology; 7. Ministry of Roads and Urban Development; 8.Ministry of Agriculture Jihad; 9. Ministry of Industry, Mine and Trade; 10.Ministry of Health and Medical Education; 11.Ministry of Petroleum; 12.Ministry of Energy; 13.Ministry of Cooperatives, Labour, and Social Welfare; 14.Ministry of Communication & Information Technology; 15.Ministry of Defence and Armed Forces Logistics; 16. Prosecutor-General of Iran; 17. Head of Inspection Organization Of Iran; 18–19. Two Members of Parliament; 20. Head of Iran Chamber of Commerce, Industries, Mines and Agriculture; 21. Head of Iran Cooperative Chamber; 22. Head of Iran Chamber of Guilds; 23–26. Four experienced experts in standard affairs; 27. Head of IRIB; 28. Head of National Qualification Center of Iran; 29. Head of the Competition Council

The computed network metrics, including degree, closeness, betweenness, and eigenvector centralities, are reported in Appendix C. The importance of nodes in information flow is measured by betweenness centrality. The highest values in Betweenness centrality are found in the Supreme Council for Standards (427.59), Supreme Council of Health and Food Security (332.45), National Council for the Elderly (218.66), and Supreme Council of Health Insurance (194). Eigen Centrality measures a node's influence in a network based on the number of linkages it has to other nodes. The Supreme Council for Youth and Sports (0.0407), the Supreme Council for Standards (0.0374), the Supreme Council for Labor and Employment (0.0315), the Supreme Council for Environmental Protection (0.0310), and the Supreme Council for Health and Food Security (0.0295) have the highest Eigen Centrality values.

**Fig. 3:**
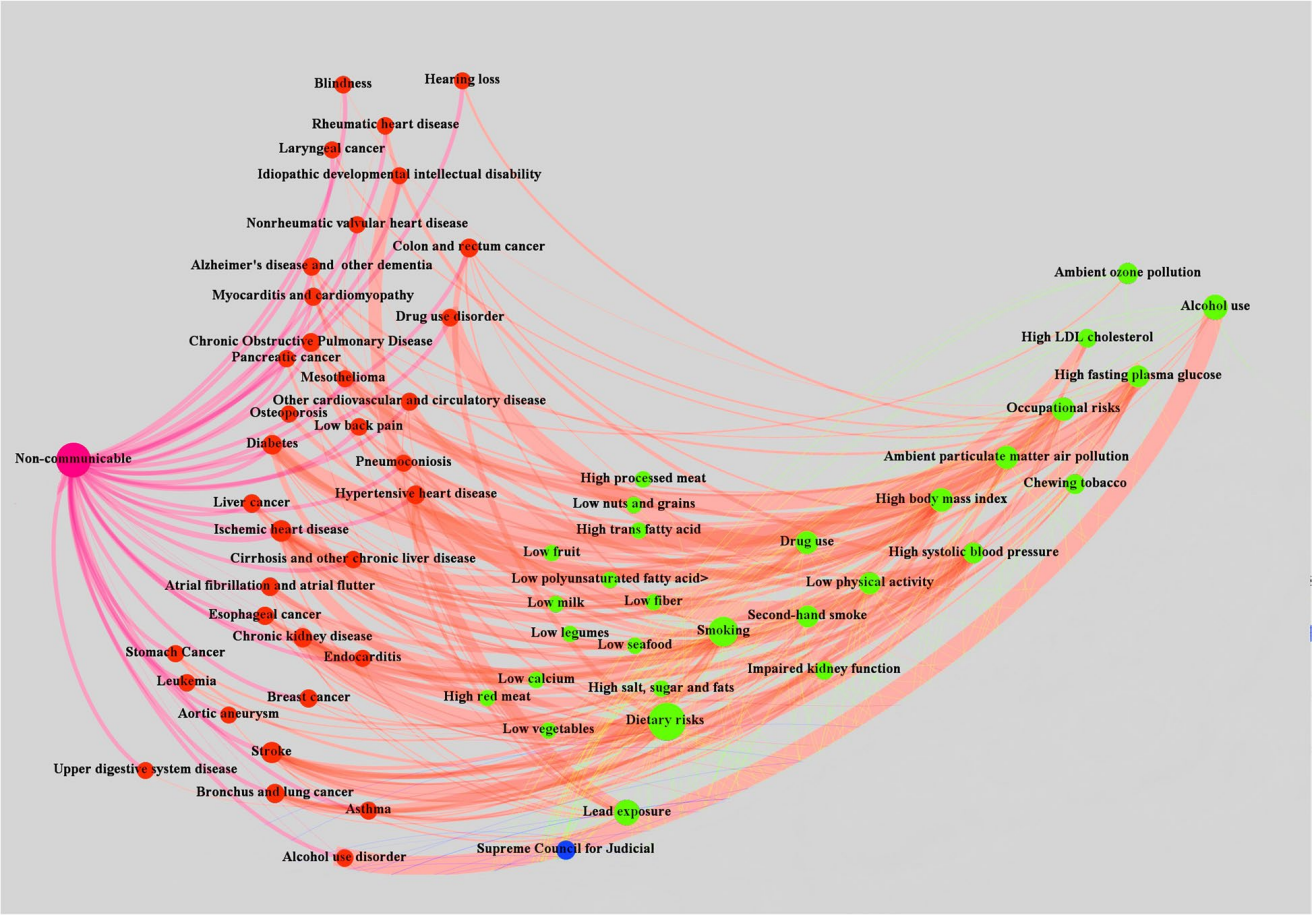
Risk factors and their contribution to the burden (DALY) of NCDs in Iran, 2017

**Fig. 4:**
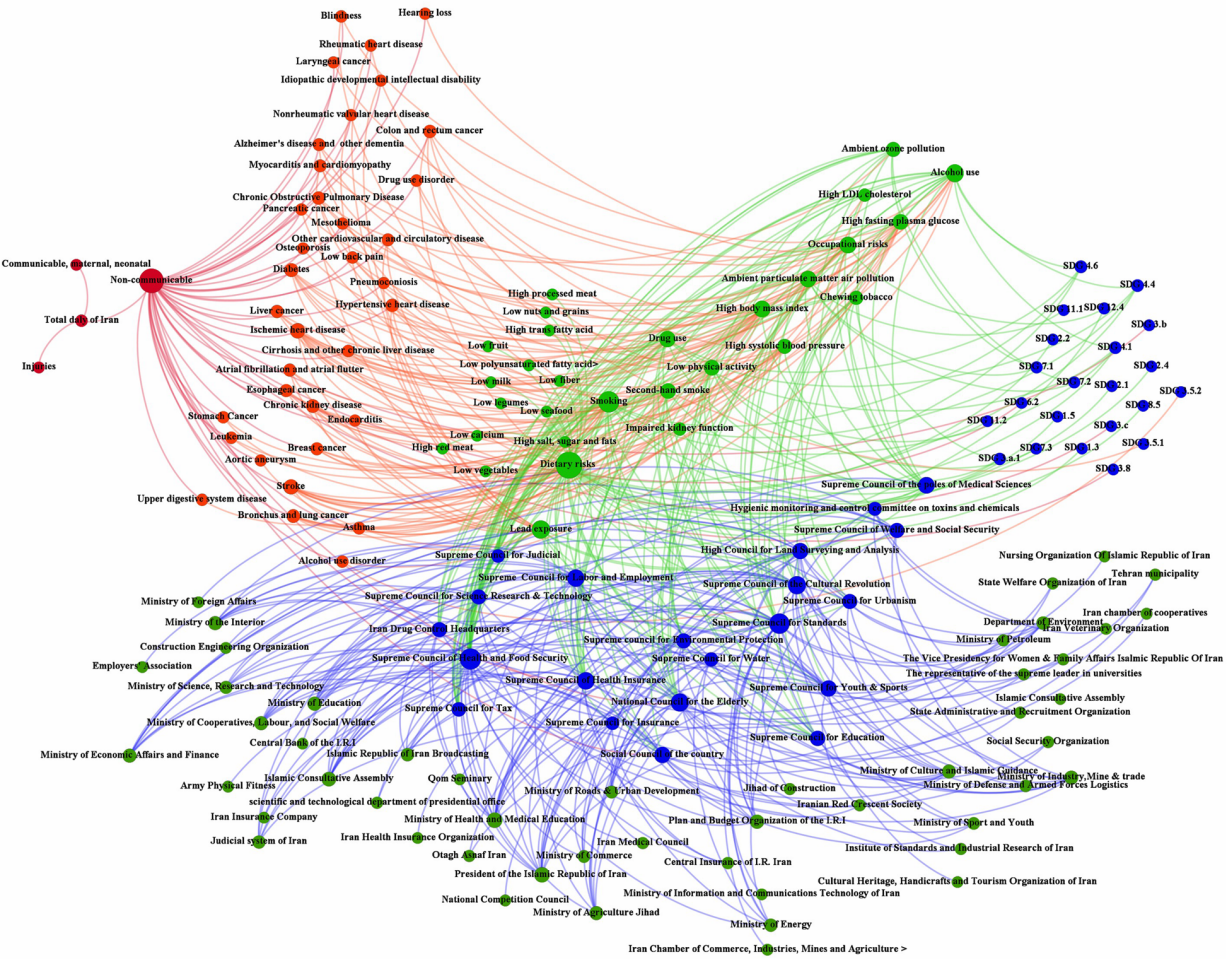
The network map of actors, councils, SDGs, risk factors and NCDs. Dark green nodes: actors including ministries, parliament, institute. Blue nodes: councils and SDG targets. Light green nodes: risk factors. Red nodes: NCDs (burden). Green edges: membership. Blue edges: the potential impact of the councils on risk factors. Green edges (center): Pathogenicity of Risk Factors. Red edges: Accumulation of noncommunicable diseases burden

Figures 3 and 4 show the network map drawn based on Table 3. The map also outlines the SDGs with the effects on the risk factors that are related to NCDs. Figure 3 shows each risk factor's contribution to the burden of disease through the edge diameter, while Fig. 4 shows all characteristics (actors, councils, SDGs, Risk Factors, and NCDs). The MoHME and the President of the Islamic Republic of Iran were identified as the most active actors on selected councils, with the out-degree value of 13. SDG 4 (Ensure inclusive and equitable quality education and promote lifelong learning opportunities for all) with out-degree value four could affect most NCD's risk factors.

Figure 3 shows the contribution of risk factors to the burden (DALY) of NCDs in Iran. The greater diameter of the edges displays a greater contribution of the risk factor to the associated disease burden.

Figure 4 shows the whole network, including members, councils, risk factors, and NCDs. The dark green represents state actors such as ministries, institutes and parliament. The blue edges show the membership of actors, and the blue nodes show the selected councils with a potential influence on the risk factors (Light green nodes). The contribution of risk factors to the burden (DALY) of NCDs (Red nodes) are also displayed with red edges.

Our findings indicate macro-level interventions required to target public policies and translate them to policymakers, aiming to design more meaningful interventions by the MoHME to enhance intersectoral legislation and policies for the prevention and control of NCDs. Our study also revealed the councils and their potential capacities for the prevention and control of NCDs, which have not been sufficiently attended by the MoHME so far.

Table 5 summarizes the proposed interventions based on the ILF model depend, drawn from the expert's opinion.

**Table 5:** Proposed interventions and their levels

Level	Proposed Intervention	Appropriateness	Feasibility
**Paradigm**	1. Continuous insistence and emphasis on health in all policies in the approaches of the Councils to attract attention and change the attitudes and values of key council members (changing the values of key individuals)	High	Medium
	2. Planning to reform and improve the organizational culture with health values in councils and members (ministries) (changing organizational values)	High	Low
	3. Frequently challenging hypotheses, values, and priorities that are harmful to health in supreme councils	High	Very low
	4. Identifying the educational and social centers in which council members were trained before reaching their positions and inject health-oriented principles into the training of these centers and communities	Medium	Low
**Goals**	1. Developing new health-related goals for supreme councils to improve community health	High	Medium
	2. Preparing the essential prerequisites for achieving the national health goals related to the responsibilities of each council and delivering them to the secretariat of the supreme councils	High	High
**System structure**	1. To membership in supreme councils with significant effect over health determinants	High	High
	2. Using weighted ballots in supreme councils with more weight of votes of the representative of the Ministry of Health in health-oriented affairs	Very High	Low
	3. Combining supreme councils (with relevant specialized committees), for example, combining councils dealing with welfare, social affairs, and community health	High	Low
	4. Expanding health secretariats in other ministries and directing them to influence representatives of relevant ministries in supreme councils	High	Medium
	5. Improving the communication structure of the representative of the Ministry of Health in the supreme councils with the management body and units, offices, and specialized centers of the MoHME	Medium	High
	6. Creating a database and developing the health-based information flow system related to the tasks and mission of each council	Medium	High
	7. Establishing inter-ministerial committees and working groups at the level of experts in the sub-councils	High	Medium
**Feedback and delays**	1. Monitoring policies and approvals of councils to respond immediately to possible undesirable approvals	Medium	Medium
	2. Creating negative feedback loops (e.g., legal and punitive action against harmful approvals)	High	Medium
	3. Defining index-based critical values to provide negative feedback on councils (Like a thermostat, it prevents the system from collapsing and keeps it within safe boundaries)	High	Medium
	4. Controlling and slowing down positive feedback loops in the relationship between councils and their members (for example, ministers use their influence on council members to advance their programs that harm public health)	High	Low
	5. Organizing lectures, meetings, and specialized publications related to councils and their field of work	Low	High
	6. Providing feedback on the effects of the activities of high councils and ministries and organizations to people and specialized communities to create sensitivity and awareness in society	Low	Medium
	7. Creating feedback loops in places that did not exist before and involving a wide range of stakeholders in feedback	Medium	High
**Structural elements**	1. Allocating a special workforce to deal with the affairs of each council in the MoHME	Medium	High
	2. Using human resources training (representative of the MoHME and following team in councils) to improve leadership skills, promote cooperation, and manage conflict of interest situations	High	High
	3. Extracting policies and inter-sectoral interventions of national health programs and network them with supreme councils	Low	High
	4. Trying to commit commitment to allocate resources to members of the councils until the commitment to goals without the operational program	Medium	Medium
	5. Prioritize public health issues related to supreme councils based on disease burden and mortality or approximate time for reform	High	High
	6. Establishing a health-oriented point in the process of reviewing, approving, and evaluating policies in councils	High	Medium

## Discussion

This research aimed to identify collective decision-making centers in the government of Iran and determine the extent to which their capacity and function can address NCDs’ related risk factors. Our study revealed that while members of each council can have a different impact on the risk factors of NCDs, the identified stakeholders and councils have different values of interest, position, power, and influence on Iran's NCDs-related policies. Based on its constitution's mission statement, the SCHFS is accountable for guiding ISC on health through its many bilateral and multilateral cooperations with relevant ministries and organizations [49].

The highest closeness centrality and betweenness centrality measures were related to the Supreme Council of Health and Food Security, the Supreme Council for Standards, and it shows the impact of this Council. Usually, when a node is important and is a network hub, not only does it has a high degree centrality but also a high centrality according to other centrality measures (betweenness, closeness). Moreover, different centrality measures have different implications. For example, closeness centrality shows access to other nodes, while eigenvector centrality shows the node's importance due to the importance of its neighbors. The highest closeness centrality and betweenness centrality measures were related to the Supreme Council of Health and Food Security and the Supreme Council for Standards, highlighting their superior impact.

Building capacity for risk-based interventions is crucial to implementing appropriate NCD policies and enhancing members' commitment to each council. Here, we will discuss the two leading councils in the prevention and control of NCDs and related risk factors in Iran:

1. The SCHFS: Its most important mission is to “coordinate and make policy on all matters related to public health and food security and nutrition”, aiming to materialize the notion of health in all policies. In other words, the Council is in charge of developing inter-sectoral collaboration between the MoHME, as the steward of the health sector, and other relevant organizations within the health system. In addition to its national structure with the president and several ministers as its members, the council has its provincial branches led by the governor of the province. The SCHFS has been enacted in various areas (Table 6).

**Table 6:**
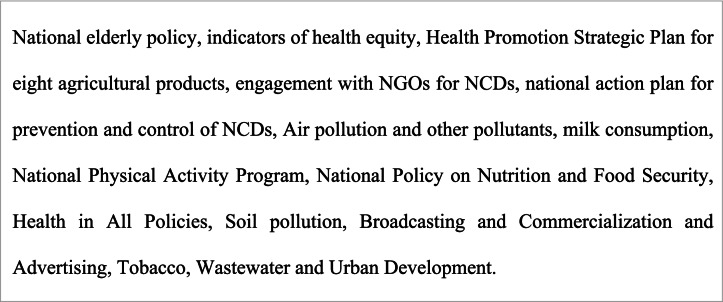
Areas enacted by the SCHFS
  The Council covers most risk factors related to NCDs and attempts to include them within relevant legislation, research, and cross-sectoral cooperation while acting to enhance awareness. The SCHFS approved the National action plan for preventing and controlling NCDs in Iran. This led to the establishment of the Iranian Non-Communicable Diseases Committee (INCDC) within the MoHME [10]. So far, to foster multisectoral collaboration through the SCHFS, the INCDC has signed 14 agreements with other ministries and national organizations for NCDs’ risk reduction.
2. The Supreme Council for Standards (SCS) is responsible for policymaking related to the National Standard Organization (NSO) of Iran. The NSO has 34 national committees to set national standards. Except for medicines and health services, all products and services should be approved by the NSO. The NSO reviews or create over 2000 standards set every year. It also assesses the compliance of goods or services with established standards. The SCS can reduce NCDs' prevalence by targeting materials, whose consumption can increase NCDs’ related risk factors (Table 4). The SCS has a greater potential to guide standard-setting policies in the NSO towards further preventive measures. The standards of products such as beverages, sauces, canned foods, juices, sugar, compote, ready meals and auto parts are also determined by this organization.
  As the MoHME has a representative in these councils, it can potentially open a window of opportunity in the Iranian administrative system towards reducing unhealthy behaviors and other NCDs’ risk factors. Based on the law, the decisions of these Councils are legally binding for everyone (ministries, organizations, the private section, etc.). The SCHFS also supports studies aiming to reform membership, voting processes, and prioritization of issues [50]. To promote public health initiatives, especially related to NCDs, it is necessary that values, skills, information systems, and organizational subsystems in these councils be reviewed and supported through the vital capacity of the MoHME's role-playing. Likewise, the SCS supports a screening framework for health impact assessment [18]. Legal and regulatory reforms’ role in the global and national response to NCDs is more significant than it has been acknowledged. Some researchers have investigated the importance of intersectoral legislation in implementing WHO's “best buys” and other recommended interventions. They pointed out that successful implementation of the preventive interventions, particularly in the LMICs, depends on the new legislation and reform of existing laws and standards [19, 36, 51]; Therefore, legislative leverage could be an effective and affordable means to control NCDs and their related risk factors.

 

Collaboratives Core dimensions of connectivity in public health collaborators such as network membership, network interaction, the health department's role, frequency of interactions, strategic value, trust, and reciprocity are critical to the success of multisectoral partnerships [11]. Our study showed that the MoHME is a member as well as leads many councils, while its vote is equal to other members for adopting policies by a majority vote of 50% + 1. As the MoHME is the guardian of population health, we advocate the weighted voting for the MoHME in the councils to help councils make better and stronger decisions for more meaningful prevention and control of NCDs [52].

The statutes of councils state that voting is based on members' opinions. Accordingly, the most important action for health-centered decisions in the Supreme Councils is to express its importance and provide health-oriented values and evidence to the council members by the MoHME representative, mostly the minister of health & medical education or his/her representative.

NCD’s Risk factors are multidimensional; for instance, unhealthy nutrition stems from several dimensions, including proximal factors (food habits, lifestyle, and lack of education) and distal factors (poverty, illiteracy, food processing industries, tax system, supply and demand system, agriculture, import and export, minimum income). This study reconfirmed that each of these dimensions could be addressed through the councils and their members to make good policies for public health. As the president is the leader of most supreme councils, s/he can act as a catalyst for multisectoral and multistakeholder actions on NCDs.

NCDs and their risk factors are complex and multidimensional; addressing their unpredictable and long-term outcomes requires heterogeneous interaction and meaningful intersectoral collaboration. Social science methods for exploring complex systems, e.g., system dynamics, agent-based modeling, and network analysis, can help measure and analyze the relationships and flows among multiple and complex actors. The literature is tiny about using these methods to better manage NCDs and relevant risk factors [39]. Our study revealed that supreme councils could facilitate the creation of evidence support, setting goals & targets, coordination, advocacy, monitoring & evaluation, policy guidance, financial support, providing legal mandate, and help better implementation & management of NCDs [34].

The proposed intervention at the paradigm and goal levels are long-term actions that will face significant resistance. At lower intervention levels, Creating a database and implementing a health-related information flow system to support each council's tasks and missions; Creating negative feedback loops; Defining index-based critical values to provide negative feedback is a balancing or reinforcing intervention [53]. Balancing this feedback loop necessitates prevention measures, early detection, and treatment initiation and completion [54], which can be accomplished through Structural Elements, Feedback, and Delays.

### Limitations

There are some limitations to this study. First, despite our efforts, some participants were unwilling to participate in the study. Another limitation of this study is the lack of consideration for informal contact (edge) among stakeholders; there also may be a conflict of interest among the identified stakeholders that have not been addressed.

## Conclusions

A wide range of stakeholders impacts the risk factors for noncommunicable diseases. We identified the key actors for each risk factor by analyzing stakeholders and their social networks. The Ministry of Health and Medical Education (MoHME) can use the ability of identified stakeholders to improve risk factor management by implementing the various (levels and types) of interventions identified in this study. The interventions recommended for the identified stakeholders have the potential to improve ISC, which is crucial for more effective prevention and management of modifiable risk factors for NCDs. Supreme councils and their members could serve as key nodes in implementing tailored inter-sectoral initiatives to address the risk factors for NCDs. While COVID-19 and the massive changes in the epidemiological transition might challenge the prioritization and budget allocation to fight against NCDs, social analysis of stakeholders will help, we envisage, focus on actors with the most influence to roll back the global movement to tackle NCDs and reach sustainable societies, in Iran and probably beyond.

## Supplementary Information

**Additional file 1: Appendix A.** We have provided the concepts of network analysis in Appendix A to assist the readers in understanding.

**Additional file 2: Appendix B.** defines the concepts of “appropriateness” and “feasibility”; the field of knowledge of experts and their numbers are also stated.

**Additional file 3: Appendix C.1.** Network and node-level metrics. **Appendix C.2.** Network metrics, including degree, closeness, betweenness, and eigenvector centralities, computed for the different risk factors.

## Data Availability

The datasets used and/or analyzed during the current study are available from the corresponding author upon a reasonable request.

## Abbreviations

NCDs: Non-Communicable Diseases
SNA: Social Network Analysis
SCHFS: Supreme Council for Health and Food Security
SCS: Supreme Council for Standards
SCIP: Supreme Council for Environmental Protection
SCHI: Supreme Council for Health Insurance
MoHME: Ministry of Health and Medical Education
ISC: Inter-Sectoral Collaboration
YLL: Years of Life Lost
DALYs: Disability-Adjusted Life Years
LMICs: Low and Middle-Income Countries
UMICs: Upper-Middle-Income-Countries
IAH: Intersectoral Action for Health
MDG: Millennium Development Goals
SDGs: Sustainable Development Goals
INCDC: Iranian Non Communicable Diseases Committee
NGO: Non-Governmental Organization

## References

[1] World Health Organization. Noncommunicable diseases country profiles 2018. World Health Organization; 2018. https://apps.who.int/iris/handle/10665/274512. License: CC BY-NC-SA 3.0 IGO.

[2] Bloom DE, Cafiero ET, Jané-Llopis E, Abrahams-Gessel S, Bloom LR, Fathima S (2011). The Global Economic Burden of Noncommunicable Diseases.

[3] Peykari N, Hashemi H, Dinarvand R, Haji-Aghajani M, Malekzadeh R, Sadrolsadat A (2017). National action plan for non-communicable diseases prevention and control in Iran; a response to emerging epidemic. J Diabetes Metab Disord.

[4] Peykari N, Hashemi H, Asghari G, Ayazi M, Janbabaei G, Malekzadeh R (2018). Scientometric study on non-communicable diseases in Iran: a review article. Iran J Public Health.

[5] Azadnajafabad S, Mohammadi E, Aminorroaya A, Fattahi N, Rezaei S, Haghshenas R, Rezaei N, Naderimagham S, Larijani B, Farzadfar F (2021). Non-communicable diseases’ risk factors in Iran; a review of the present status and action plans. J Diabetes Metab Disord.

[6] Murray CJ, Aravkin AY, Zheng P, Abbafati C, Abbas KM, Abbasi-Kangevari M, Abd-Allah F, Abdelalim A, Abdollahi M, Abdollahpour I, Abegaz KH. Global burden of 87 risk factors in 204 countries and territories, 1990–2019: a systematic analysis for the Global Burden of Disease Study 2019. The Lancet. 2020;17;396(10258):1223-49. 10.1016/S0140-6736(20)30752-2 PMC7566194 33069327

[7] Bloom DE, Cafiero E, Jané-Llopis E, Abrahams-Gessel S, Bloom LR, Fathima S (2012). The global economic burden of noncommunicable diseases Program on the Global Demography of Aging.

[8] Takian A, Bakhtiari A, Ostovar A (2020). Universal health coverage for strengthening prevention and control of noncommunicable diseases in COVID-19 era. Med J Islam Repub Iran..

[9] World Health Organization. WHO package of essential noncommunicable (‎PEN)‎ disease interventions for primary health care. World Health Organization; ‎2020‎. https://apps.who.int/iris/handle/10665/334186. License: CC BY-NC-SA 3.0 IGO.

[10] Amerzadeh M, Salavati S, Takian A, Namaki S, Asadi-Lari M, Delpisheh A (2020). Proactive agenda setting in creation and approval of national action plan for prevention and control of non-communicable diseases in Iran: the use of multiple streams model. J Diabetes Metab Disord.

[11] Varda DM, Chandra A, Stern SA, Lurie N (2008). Core dimensions of connectivity in public health collaboratives. J Public Health Manag Pract.

[12] Giles-Corti B, Vernez-Moudon A, Reis R, Turrell G, Dannenberg AL, Badland H (2016). City planning and population health: a global challenge. Lancet.

[13] Mialon M, Swinburn B, Sacks G (2015). A proposed approach to systematically identify and monitor the corporate political activity of the food industry with respect to public health using publicly available information. Obes Rev.

[14] World Health Organization (2013). Regional Office for Africa Multi-stakeholder dialogue on risk factors for non-communicable diseases.

[15] Hospedales CJ, Jane-Llopis E (2011). A multistakeholder platform to promote health and prevent noncommunicable diseases in the region of the Americas: the Pan American Health Organization partners forum for action. J Health Commun..

[16] Pearlman PC, Vinson C, Singh T, Stevens LM, Kostelecky B (2016). Multi-stakeholder partnerships: breaking down barriers to effective cancer-control planning and implementation in low-and middle-income countries. Sci Dipl..

[17] Rakotoniaina AL (2018). How to increase fruit and vegetable consumption: a multistakeholder approach for improved health outcomes—a report from the alliance for food & health. J Am Coll Nutr..

[18] Ross C, Orenstein M, Botchway N. Health impact assessment in the United States. New York: Springer; 2014.

[19] Magnusson RS, Patterson D (2014). The role of law and governance reform in the global response to non-communicable diseases. Global Health.

[20] Wilkin D, Hallam L, Doggett M-A (1992). Measures of need and outcome for primary health care.

[21] UN (2009). Millennium Development Goals Report 2009 United Nations Publications.

[22] Dodds F. Multi-Stakeholder Partnerships: Making Them Work for the Post-2015 Development Agenda. New York: ECOSOC/United Nations; 2015.

[23] World Health Organization‎. WHO global coordination mechanism on the prevention and control of noncommunicable diseases: final report: WHO GCM/NCD working group on the alignment of international cooperation with national NCD plans (‎Working group 3.2, 2016–2017)‎. World Health Organization; 2018. https://apps.who.int/iris/handle/10665/312273. License: CC BY-NC-SA 3.0 IGO.

[24] Collins T, Mikkelsen B, Adams J, Chestnov O, Evans T, Feigl A (2018). Addressing NCDs: a unifying agenda for sustainable development. Glob Public Health.

[25] World Health Organization. (WHO). Health in 2015: From Millennium Development Goals (MDGs) to Sustainable Development Goals (SDGs). Geneva; 2019.

[26] Bostock M. Zoomable Circle Packing/D3/Observable. 2018. Available: https://observablehq.com/@d3/zoomable-circle-packing. [online]

[27] Raum S (2018). A framework for integrating systematic stakeholder analysis in ecosystem services research: Stakeholder mapping for forest ecosystem services in the UK. Ecosyst Serv.

[28] Reed MS, Graves A, Dandy N, Posthumus H, Hubacek K, Morris J (2009). Who's in and why? a typology of stakeholder analysis methods for natural resource management. J Environ Manage.

[29] Hunter RF, Wickramasinghe K, Erguder T, Bolat A, Ari HO, Yildirim HH (2019). National action plans to tackle NCDs: role of stakeholder network analysis. BMJ.

[30] Kadushin C (2012). Understanding social networks: Theories, concepts, and findings.

[31] Carrington PJ, Scott J, Wasserman S, editors. Models and methods in social network analysis. Cambridge University Press; 2005.

[32] Watkins C, Massey D, Brooks J, Ross K, Zellner ML. Understanding the mechanisms of collective decision making in ecological restoration: an agent-based model of actors and organizations. Ecol. Soc. 2013;18(Article 2). 10.5751/ES05497-180232.

[33] The Organization and Institution of the Presidency, Administrative Councils (2011). A Legal Review of the Position and Status of Councils in Iranian Structure.

[34] World Health Organization (2012). Intersectoral governance for health in all policies: structures, actions and experiences: World Health Organization. Regional Office for Europe.

[35] Bastian M, Heymann S, Jacomy M (2009). Gephi: an open source software for exploring and manipulating networks Third international AAAI conference on weblogs and social media.

[36] Kaldor JC, Thow AM, Schönfeldt H (2019). Using regulation to limit salt intake and prevent non-communicable diseases: lessons from South Africa’s experience. Public Health Nutr.

[37] Bakhtiari A, Takian A, Majdzadeh R, Haghdoost AA (2020). Assessment and prioritization of the WHO “best buys” and other recommended interventions for the prevention and control of non-communicable diseases in Iran. BMC Public Health.

[38] WHO Global action plan for the prevention and control of noncommunicable diseases 2013–2020. Geneva: World Health Organization; 2013. http://apps.who.int/iris/bitstream/10665/94384/1/9789241506236_eng.pdf?ua=1. Accessed 15 Jan 2021.

[39] Nianogo RA, Arah OA (2015). Agent-based modeling of noncommunicable diseases: a systematic review. Am J Public Health.

[40] Mogalakwe M (2006). The use of documentary research methods in social research. African Sociological Review/Revue Africaine De Sociologie.

[41] Scott JJSR (1990). A matter of Record: Documentary Sources.

[42] World Health Organization (2016). World health statistics 2016: monitoring health for the SDGs sustainable development goals: World Health Organization.

[43] Griggs D, Nilsson M, Stevance A, McCollum D (2017). A guide to SDG interactions: from science to implementation.

[44] Nilsson M. Understanding and mapping important interactions among SDGs: Readying institutions and policies for integrated approaches to implementation of the 2030 Agenda., in: Expert Meet. Prep. HLPF 2017. 2016. pp. 1–33. https://sustainabledevelopment.un.org/content/documents/12067Understanding and mapping important interactions among SDGs.pdf.

[45] Weitz N, Carlsen H, Nilsson M, Skanberg K (2018). Towards systemic and contextual priority setting for implementing the 2030 Agenda. Sustain Sci.

[46] Moinuddin M (2017). Sustainable Development Goals Interlinkages and Network Analysis: A practical tool for SDG integration and policy coherence: Institute for Global Environmental Strategies.

[47] Mislove A, Marcon M, Gummadi KP, Druschel P, Bhattacharjee B. Measurement and analysis of online social networks. In Proceedings of the 7th ACM SIGCOMM conference on internet measurement - IMC ’07 (p. 29). New York: ACM Press; 2007. http://dl.acm.org/citation.cfm?id=1298306.1298311.

[48] Johnston LM, Matteson CL, Finegood DT (2014). Systems science and obesity policy: a novel framework for analyzing and rethinking population-level planning. Am J Public Health.

[49] Damari B, Heidari A. Implementation of integrated management of non-communicable disease prevention and control in Iran: A proposal. Payesh (Health Monitor). 2020;15;19(1):7-17.

[50] Damari B, Vosoogh-Moghaddam A, Riazi-Isfahani S (2018). Implementing health impact assessment at national level: an experience in Iran. Iran J Public Health.

[51] Gostin LO, Abou-Taleb H, Roache SA, Alwan A (2017). Legal priorities for prevention of non-communicable diseases: innovations from WHO’s Eastern Mediterranean Region. Public Health.

[52] Houy N, Zwicker WS (2014). The geometry of voting power: weighted voting and hyper-ellipsoids. Games Econ Behav.

[53] Meadows DH. Thinking in systems: a primer. Chelsea Green. 2008.

[54] Durham J, Schubert L, Vaughan L, Willis CD (2018). Using systems thinking and the Intervention Level Framework to analyse public health planning for complex problems: Otitis media in Aboriginal and Torres Strait Islander children. PLoS One..

