# Supplementary material for: Intersectoral collaboration in the management of non-communicable disease’s risk factors in Iran: stakeholders and social network analysis
Source: BMC Public Health. 2022 Sep 2;22:1669. doi: 10.1186/s12889-022-14041-8 (PMC9439719; doi:10.1186/s12889-022-14041-8)
Supplement: Supplementary file 1 — Additional file 1: Appendix A. We have provided the concepts of network analysis in Appendix A to assist the readers in understanding. [file 12889_2022_14041_MOESM1_ESM.docx]

Appendix A:

We have provided the concepts of network analysis in Appendix A to assist the readers in understanding.

| Key Terms For Network Analysis | |
| --- | --- |
| The density of a graph | The density of a graph is a measure of how close the graph is to a complete graph with the same number of nodes. It is defined as the ratio of the total number of edges present in a graph to the total number of edges possible in the graph. |
| Closeness centrality | The closeness centrality of a node measures how accessible every other node in the graph is from the considered node. It is defined as the inverse of the sum of the shortest distances of every other node in the network from the current node. Closeness centrality is an indicator of the speed at which information will be transfused into the network, starting from the current node. |
| Betweenness centrality | Betweenness Centrality measures the level at which any given node serves as a bridge connecting other nodes. |
| Eigenvector centrality | The basic concept behind the eigenvector centrality is that a node with connections to more influential nodes is considered to be more influential than those connected to less influential nodes. |
| Average degree | The average degree represents the average number of unweighted  connections across a network |
| Average weighted degree | To find the average weighted degree for a graph with weighted edges, Gephi computes the average mean of the sum of the weights of the incident edges on all the nodes in the graph. |
| Clustering coefficient | the clustering coefficient of a node is the ratio of existing links connecting a node's neighbors to each other to the maximum possible number of such links. The clustering coefficient for the entire network is the average of the clustering coefficients of all the nodes. A high clustering coefficient for a network is another indication of a small world |
| Average clustering coefficient | The Average Clustering Coefficient is the mean value of individual coefficients. |
| Diameter | The diameter of a network refers to the length of the longest of all the computed shortest paths between all pairs of nodes in the network. |
