# Supplementary material for: Intersectoral collaboration in the management of non-communicable disease’s risk factors in Iran: stakeholders and social network analysis
Source: BMC Public Health. 2022 Sep 2;22:1669. doi: 10.1186/s12889-022-14041-8 (PMC9439719; doi:10.1186/s12889-022-14041-8)
Supplement: Supplementary file 2 — Additional file 2: Appendix B. defines the concepts of “appropriateness” and “feasibility”; the field of knowledge of experts and their numbers are also stated. [file 12889_2022_14041_MOESM2_ESM.docx]

**Appendix B.1**

Appendix B defines the concepts of "appropriateness" and "feasibility"; the field of knowledge of experts and their numbers are also stated.

Definition of the words "appropriateness" and "feasibility."

| Appropriateness  Very low: The appropriateness of the policy option in solving the real-world problem is very low.  Low: The appropriateness of the policy option in solving the real-world problem is low.  Medium: The appropriateness of the policy option in solving the real-world problem is moderate.  High: The appropriateness of the policy option in solving the real-world problem is high.  Very High: The appropriateness of the policy option in solving the real-world problem is too great. |
| --- |
| Feasibility:  This means that from various dimensions (technical, required resources, structural, social, etc.), the proposed policy option is likely to become operational.  possibility:  Very low: Impossible impractical  Low: Possibility to do low  Medium: Somewhat possible  High: Possible / practical  Very High: Definitely possible, Definitely practical |

Appendix B.2

B.2 Experts interviewed

|  | Position | Number |
| --- | --- | --- |
| 1 | University, Academic Board, Ministry of Health | 5 |
| 2 | General Practitioner, Medical Council | 3 |
| 3 | insurance experts | 2 |
| 4 | Academic, Health Policy | 3 |
| 5 | University, Academic, Center for Social Determinants of Health | 20 |
