# Supplementary material for: Intersectoral collaboration in the management of non-communicable disease’s risk factors in Iran: stakeholders and social network analysis
Source: BMC Public Health. 2022 Sep 2;22:1669. doi: 10.1186/s12889-022-14041-8 (PMC9439719; doi:10.1186/s12889-022-14041-8)
Supplement: Supplementary file 3 — Additional file 3: Appendix C.1. Network and node-level metrics. Appendix C.2. Network metrics, including degree, closeness, betweenness, and eigenvector centralities, computed for the different risk factors. [file 12889_2022_14041_MOESM3_ESM.docx]

**Appendix C.1. Network and node-level metrics**

| **Parameter** | **value** |
| --- | --- |
| Nodes | 161 |
| Edges | 515 |
| Density | 0.02 |
| Average degree | 3.199 |
| Average weighted degree | 78.38 |
| Clustering Coefficient | 0.0059 |
| Average clustering coefficient (directed)* | 0.002 |
| Diameter | 5 |

*The Average Clustering Coefficient is the mean value of individual coefficients

**Appendix C.2. Network metrics, including degree, closeness, betweenness, and eigenvector centralities, computed for the different risk factors**

| **Type** | **Node** | **Degree** | **In degree** | **Out degree** | **Weighted out-degree** | **Closeness**  **centrality** | **Betweenness**  **centrality** | **Eigenvector**  **centrality** | **Hub** |
| --- | --- | --- | --- | --- | --- | --- | --- | --- | --- |
| **Risk factors** | Ambient particulate matter air pollution | 17 | 12 | 5 | 95.79 | 0.7 | 62.8609 | 0.0993 | 0.0 |
|  | Ambient ozone pollution | 11 | 10 | 1 | 8.16 | 0.5 | 11.54 | 0.1050 | 0.0 |
|  | Lead exposure | 22 | 10 | 12 | 136.82 | 0.82 | 336.64 | 0.0953 | 0.0 |
|  | Occupational risks | 17 | 9 | 8 | 257.66 | 0.77 | 257.88 | 0.1009 | 0.0 |
|  | Smoking | 32 | 16 | 16 | 240.4 | 0.85 | 502.40 | 0.1529 | 0.0 |
|  | Chewing tobacco | 9 | 8 | 1 | 9.56 | 0.5 | 10.28 | 0.0836 | 0.0 |
|  | Second-hand smoke | 14 | 8 | 6 | 36.02 | 0.73 | 56.06 | 0.0911 | 0.0 |
|  | Alcohol use | 21 | 15 | 6 | 116.7 | 0.73 | 196.47 | 0.1509 | 0.0 |
|  | Drug use | 16 | 13 | 3 | 150.76 | 0.62 | 98.56 | 0.1279 | 0.0 |
|  | Dietary risks (including 13 risks: Low fruit, vegetables, legumes, nuts and grains, milk, fiber, calcium, seafood, polyunsaturated fatty acid and High salt, sugar and fats, red meat, processed meat, trans fatty acid) | 50 | 39 | 11 | 222.65 | 0.81 | 663.94 | 0.2505 | 0.0 |
|  | Low physical activity | 14 | 10 | 4 | 32.46 | 0.66 | 52.68 | 0.1077 | 0.0 |
|  | High systolic blood pressure | 11 | 3 | 8 | 370.79 | 0.76 | 40.34 | 0.0371 | 0.0 |
|  | High fasting plasma glucose | 14 | 4 | 10 | 266.51 | 0.8 | 62.34 | 0.0487 | 0.0 |
|  | High LDL cholesterol | 6 | 4 | 2 | 71.87 | 0.57 | 5.80 | 0.0487 | 0.0 |
|  | High body mass index | 18 | 4 | 14 | 348.79 | 0.84 | 123.16 | 0.0487 | 0.0 |
|  | Impaired kidney function | 6 | 3 | 3 | 120.59 | 0.62 | 6.99 | 0.0339 | 0.0 |
| **councils** | Supreme Council of Health and Food Security | 32 | 15 | 17 | 1360 | 0.596 | 332.45 | 0.0295 | 0.3998 |
|  | Supreme Council for Justice | 7 | 1 | 6 | 120 | 0.518 | 8.61 | 0.0019 | 0.1897 |
|  | Supreme Council for Centers of Excellence in Medical Sciences | 17 | 2 | 15 | 900 | 0.565 | 48.66 | 0.0039 | 0.3552 |
|  | Supreme Council for Youth & Sports | 16 | 13 | 3 | 210 | 0.5 | 98.66 | 0.0407 | 0.0972 |
|  | Supreme Council for Standards | 29 | 19 | 10 | 800 | 0.541 | 427.59 | 0.0374 | 0.2702 |
|  | Supreme Council for Water | 9 | 8 | 1 | 40 | 0.466 | 7.17 | 0.0157 | 0.0506 |
|  | Supreme Council for Education | 13 | 5 | 8 | 160 | 0.539 | 43.40 | 0.0098 | 0.2496 |
|  | Supreme Council for Insurance | 7 | 6 | 1 | 80 | 0.466 | 20.68 | 0.0118 | 0.0506 |
|  | Supreme Council of Health Insurance | 19 | 9 | 10 | 800 | 0.550 | 194 | 0.0177 | 0.2696 |
|  | Supreme Council of the Cultural Revolution | 22 | 14 | 8 | 400 | 0.539 | 158.02 | 0.0276 | 0.2293 |
|  | Supreme Council for Science Research & Technology | 12 | 8 | 4 | 160 | 0.509 | 74.17 | 0.0157 | 0.0868 |
|  | Supreme council for Environmental Protection | 12 | 7 | 5 | 400 | 0.516 | 80.13 | 0.0310 | 0.1374 |
|  | Supreme Council of Welfare and Social Security | 12 | 11 | 1 | 60 | 0.466 | 14.58 | 0.02170 | 0.0506 |
|  | Supreme Council for Labor and Employment | 19 | 16 | 3 | 90 | 0.457 | 138.61 | 0.0315 | 0.0701 |
|  | Supreme Council for Tax | 10 | 1 | 9 | 360 | 0.540 | 12.33 | 0.0019 | 0.2726 |
|  | Supreme Council for Urbanism | 12 | 8 | 4 | 160 | 0.511 | 58.63 | 0.0157 | 0.0903 |
|  | High Council for Land Preparation and Analysis | 16 | 15 | 1 | 1 | 0.466 | 46.52 | 0.0295 | 0.0506 |
|  | National Council for the Elderly | 21 | 12 | 9 | 450 | 0.542 | 218.66 | 0.0236 | 0.2481 |
|  | Social Council of the country | 15 | 11 | 4 | 240 | 0.508 | 94.98 | 0.0217 | 0.1735 |
|  | Iran Drug Control Headquarters | 12 | 7 | 5 | 350 | 0.518 | 25.94 | 0.0138 | 0.1533 |
|  | Hygienic monitoring and control committee on toxins and chemicals | 8 | 5 | 3 | 210 | 0.5 | 44.09 | 0.0098 | 0.0901 |
